# Stereo-selective conversion of mandelonitrile to (*R*)-(−)-mandelic acid using immobilized cells of recombinant *Escherichia coli*

**DOI:** 10.1007/s13205-012-0058-4

**Published:** 2012-03-29

**Authors:** Sandip V. Pawar, Vachan Singh Meena, Shubhangi Kaushik, Ashwini Kamble, Sandeep Kumar, Yusuf Chisti, U. C. Banerjee

**Affiliations:** 1 Department of Pharmaceutical Technology (Biotechnology), National Institute of Pharmaceutical Education and Research, Sector-67, SAS Nagar, 160 062 Punjab, India; 2 School of Engineering, Massey University, Private Bag 11 222, Palmerston North, New Zealand

**Keywords:** Nitrilase, Mandelic acid, Mandelonitrile, Packed bed reactor, Immobilized cells

## Abstract

Immobilized cells of a recombinant *Escherichia coli* expressing nitrilase from *Pseudomonas putida* were used to catalyze the hydrolysis of mandelonitrile (2-hydroxy-2-phenylacetonitrile) to (*R*)-(−)-mandelic acid. The cells had been immobilized by entrapment in an alginate matrix. Conditions for the hydrolysis reaction were optimized in shake flasks and in a packed bed reactor. In shake flasks the best conditions for the reaction were a temperature of 40 °C, pH 8, biocatalyst bead diameter of 4.3 mm, sodium alginate concentration in the gel matrix of 2 % (w/v, g/100 mL), a cell dry mass concentration in the bead matrix of 20 mg/mL, an initial substrate concentration of 50 mM and a reaction time of 60 min. Under these conditions, the conversion of mandelonitrile was nearly 95 %. In the packed bed reactor, a feed flow rate of 20 mL/h at a substrate concentration of 200 mM proved to be the best at 40 °C, pH 8, using 4.3 mm beads (2 % w/v sodium alginate in the gel matrix, 20 mg dry cell concentration per mL of gel matrix). This feed flow rate corresponded to a residence time of 0.975 h in the packed bed.

## Introduction

The conventional harsh chemical methods are extensively substituted by environment-friendly biocatalysts for the synthesis of pharmaceutically important intermediates (Kumar et al. 2010). The interest towards use of nitrilase has been tremendously increasing since past decade leading to the easy hydrolysis of nitriles (Martinkova and Mylerova 2003; Chen et al. 2008; Kaplan et al. 2006; Rustler et al. 2007); it offers significant advantages over other routes as cheap starting material, absence of cofactor involvement and the possibility of carrying reactions under mild condition (Banerjee et al. 2002; He et al. 2010; He et al. 2007). Additionally, the existence of nitrile-hydrolyzing enzymes that show *enatio*- and *regio*-selectivity offers synthetic possibilities that are difficult to achieve by conventional catalytic approaches (Naik et al. 2008; Kiziak et al. 2005). Immobilized whole cells of microorganisms are widely used as catalysts for various biotransformation processes (Bucko et al. 2005; Niladevi and Prema 2008). When applicable, whole cell biocatalysis avoids the need to extract and purify intracellular enzymes and is therefore less expensive compared with immobilized enzyme catalysts (Givry et al. 2008; Takeru et al. 2005).

(*R*)-(−)-mandelic acid is an important pharmaceutical intermediate for the production of semi-synthetic cephalosporins, penicillins (Terreni et al. 2001; Tang et al. 2009) antitumor agents (Surivet and Vatele 1998) and antiobesity drugs (Mills et al. 1983). Its derivatives are used also as chiral resolving agents (Kinbara et al. 1996; Xue et al. 2011). Polymers of mandelic acid have been developed into antimicrobial contraceptives for vaginal prophylaxis (Zaneveld et al. 2002). Derivatives of mandelic acid have been shown to have antifungal activity (Kope et al. 1991), broad spectrum *β*-lactamase inhibitor activity (Mollard et al. 2001) and anti-oxidative properties (Ley and Bertram 2001).

**Fig. 1:**
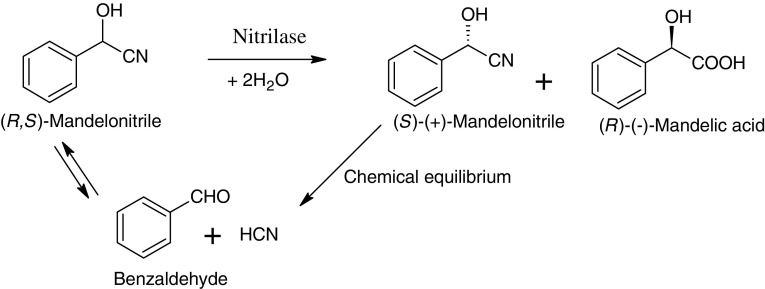
Reaction scheme for enantioselective hydrolysis of (*R*,*S*)-mandelonitrile for synthesis of (*R*)-mandelic acid

Mandelic acid is produced by chemical as well as enzymatic methods; several approaches to obtain enatiomerically pure mandelic acid have been reported in the literature (Yadav and Sivakumar 2004; Xiao et al. 2005, 2008; Oda et al. 1992; Patterson et al. 1981; Miyamoto and Ohta 1992). Nitrilase-mediated production of (*R*)-(−)-mandelic acid has significant advantage over chemical methods of production; it offers excellent enatioselectivity and above all possibility of carrying out dynamic kinetic resolution which provides 100 % theoretical yield of the product (Yamamoto et al. 1991; Banerjee et al. 2006; Singh et al. 2005; Xue et al. 2011) (Fig. 1). In recent years, the hydrolysis of mandelonitrile has been reported using recombinant nitrilase from *Pseudomonas fluorescens* EBC191 (Bucko et al. 2005), *Alcaligenes faecalis* ATCC8750 (Rey et al. 2004), newly isolated nitrilase producer *Alcaligenes* sp. ECU0401 (He et al. 2007; He et al. 2010) and *A. faecalis* ZJUTB10 (Xue et al. 2011). This study reports the use of immobilized whole cells of the recombinant *Escherichia coli* BL21 (DE3) for producing (*R*)-(−)-mandelic acid from mandelonitrile in shake flask and packed bed reactor. The recombinant bacterium used harbored the nitrilase gene from *P. putida* MTCC 5110 in the IPTG-inducible plasmid pET 21 b (+) expression vector. *P. putida* was reported as an ideal candidate for stereo-selective hydrolysis of mandelonitrile, considering its higher growth rate, higher reaction rate and higher stability as compared with other nitrilase-producing microorganisms. *P. putida* nitrilase attains comparable specific activity within a shorter period of time and the fermentation time was also reported to be lower for biocatalyst generation (Kaul et al. 2004).

## Materials and methods

### Chemicals

Mandelonitrile, sodium alginate, glutaraldehyde (GA) and polyethyleneimine (PEI) were purchased from Sigma-Aldrich Chemical Co. (Milwaukee, USA). Growth media components were obtained from Hi-Media Inc. (Mumbai, India). Solvents, buffer salts, inorganic salts and other chemicals used were of analytical grade.

### Microorganism and cultivation conditions

Recombinant *E. coli* BL21 (DE3) was used for the present study. The bacterium harbored the nitrilase gene of *P. putida* MTCC 5110 and had been cloned in our laboratory (Genbank accession no. EF467660) (Banerjee et al. 2009). The stock culture was maintained on plates of Luria–Bertani agar supplemented with ampicillin. The microorganism was initially grown in shake flasks (37 °C, 200 rpm) for 16 h in a medium of the following composition: yeast extract (10 g/L), tryptone (16 g/L) and sodium chloride (5 g/L). After 16 h, 10 % (v/v) of the culture was inoculated into fresh flasks containing the aforementioned specified medium. The inducer isopropyl-*β*-d-1-thiogalactopyranoside (IPTG) was added to each flask after 1 h of inoculation and the final IPTG concentration in the flask was 1 mM. Cells were harvested after 4 h by centrifugation at 10,000*g* (4 °C) and washed with Tris–HCl buffer (100 mM, pH 7) before use.

### Immobilization of whole cells

Washed cells harvested as specified above, were immobilized by entrapment in a sodium alginate matrix (Varma and Gaikwad 2010; Givry et al. 2008). For the entrapment, 200 mg (dry weight basis) of cells was resuspended in 10 mL of Tris–HCl buffer (100 mM, pH 7). This slurry was added to a 2 % (w/v, g/100 mL) solution of sodium alginate (prepared in Tris–HCl buffer, 100 mM, pH 7) with vigorous stirring to ensure complete mixing. The resulting suspension was added dropwise via a syringe to an aqueous solution of CaCl_2_ (0.2 M). Ca-alginate beads thus formed were allowed to stand in CaCl_2_ solution for 1 h for hardening. The hardened beads were washed with distilled water and used for the biotransformation reaction.

### Biotransformation conditions

Immobilized cells (200 mg dry weight basis) were suspended in 10 mL of Tris–HCl buffer (100 mM, pH 7). The final concentration of immobilized cells was 20 mg/mL. Mandelonitrile is not soluble in aqueous solvent (sparingly soluble); hence 10 % (v/v) methanol was used as a co-solvent to make it soluble (Kaul and Banerjee 2008). It was added to an initial concentration of 30 mM in reaction mixture. The mixture was incubated in shake flasks held at 40 °C, 200 rpm, for 3 h. Bead-free samples were taken at regular intervals and analyzed by HPLC.

### Analytical methods

The concentration of mandelic acid formed in the reaction mixture was measured by high-performance liquid chromatography (Shimadzu 10AD VP, Kyoto, Japan). Mandelic acid, mandelamide and mandelonitrile were assayed on a LiChroCART^®^ C_18_ column (250 × 4 mm, 5 μm) (Merck, Darmstadt, Germany). The elution solvent was a mixture of water, methanol and phosphoric acid (59.9:40:0.1, by volume). The solvent flow rate was 0.8 mL/min. The retention times of mandelamide, mandelic acid and mandelonitrile were 4.4, 6.1 and 10.2 min, respectively. Elution was monitored by measuring the absorbance at 210 nm.

The optical purity of mandelic acid was determined by analyzing the enantiomers on Chiralcel OD-H column (250 × 0.46 mm, 5 μm) (Diacel Chemical Industries, New Jersey, USA) at a flow rate of 0.5 mL/min with a mobile phase of hexane, isopropanol and tri-fluroacetic acid (90:10:0.2, by volume). The retention times for (*S*)-(+)-isomer and (*R*)-(−)-isomer were 15.5 and 17.5 min, respectively. Absorbance was monitored at 254 nm. Enantiomeric excess (ee, %) was calculated as follows: ee = |*f*_R _− *f*_S_| × 100, where *f*_R_ and *f*_S_ are the mole fractions of the *R* and *S* enantiomers in the product such that *f*_R_ + *f*_S_ = 1.

## Results and discussion

### Effect of alginate concentration

In separate experiments, the whole cells of recombinant *E. coli* at a dry mass concentration of 20 mg/mL were immobilized using various concentrations of sodium alginate (1.0–2.5 % w/v, g/100 mL). The nominal bead size was 4.3 mm. The biocatalyst beads made from 2 % sodium alginate effectively entrapped the cells, was structurally stable and afforded a good conversion of the substrate. The beads formed at a higher concentration of sodium alginate were satisfactory in every respect, but were not as good at converting the substrate as the aforementioned beads. This was likely because the harder gels formed at sodium alginate concentrations of >2 % reduced the diffusion of the substrate in the beads. Due to the liable nature of nitrilase, milder methods are generally used for the immobilization of whole cells/enzymes. The potential adverse effects from the matrix or reagents used are less in the entrapment method of the cell immobilization. It has been reported in the literature (Panova et al. 2007; Ben-Bassat et al. 2008) that by encapsulation of whole cells, there were effective recovery and recycle of the enzyme in the case of glycolic acid production. In view of this, cells immobilized at an alginate concentration of 2 % were used in all subsequent experiments, unless stated otherwise.

### Effect of bead size

Immobilized biocatalysts commonly experience mass transfer limitations. In such cases, efficacy of catalysis declines as the size of the catalyst bead is increased (Won et al. 2005). Therefore, the choice of the bead size is important. In this study, alginate beads of various nominal sizes (3.5, 4.3, 4.8, 5.3 and 5.7 mm) were produced by controlling the drop size from the syringe. Preliminary studies with these beads showed the percent conversion of the substrate to decrease with increasing size of beads for otherwise identical conditions. Therefore, a bead size of 4.3 mm was used in all subsequent works as this size afforded a conversion value that was similar to the conversion attained with 3.5 mm beads, but the larger beads were easier to handle.

### Effect of cell loading

**Fig. 2:**
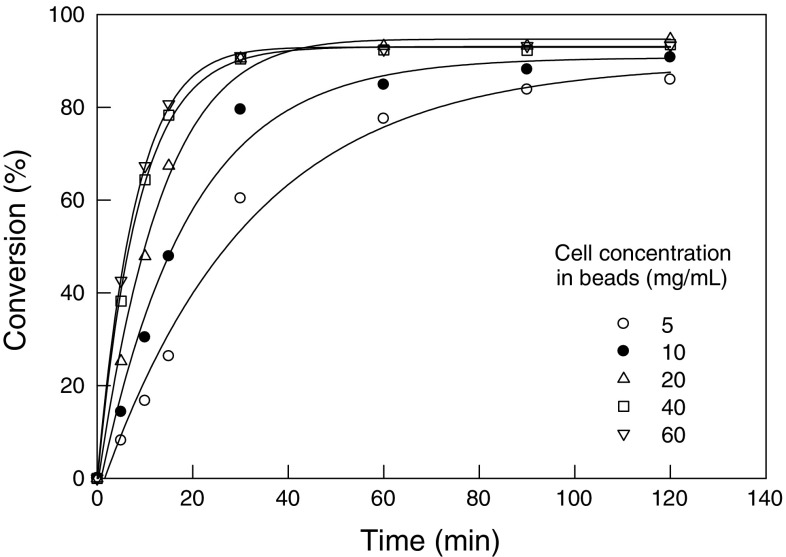
Effect of immobilized cell concentration on the conversion of mandelonitrile

The effect of cell loading in the alginate beads on their biocatalytic performance was examined. The reaction was carried out at an initial substrate concentration of 30 mM. The other conditions were a pH of 7 and 37 °C. The bead size was always 4.3 mm and the beads had been formed by suspending the specified concentration of cells in a 2 % solution of sodium alginate. Cell loading values (dry weight concentration) of 5, 10, 20, 40 and 60 mg/mL were tested. The biocatalytic activity increased with the increasing concentration of cells up to 20 mg/mL (Fig. 2). Higher cell concentrations did not further increase activity likely because the cells interfered with the diffusion of the substrate into the beads. With a cell concentration of 20 mg/mL, the conversion did not further increase after about 60 min of reaction (Fig. 2). In subsequent work, therefore, the reaction time was fixed at 60 min and the cell concentration of 20 mg/mL was used.

### Effects of pH and temperature

**Fig. 3:**
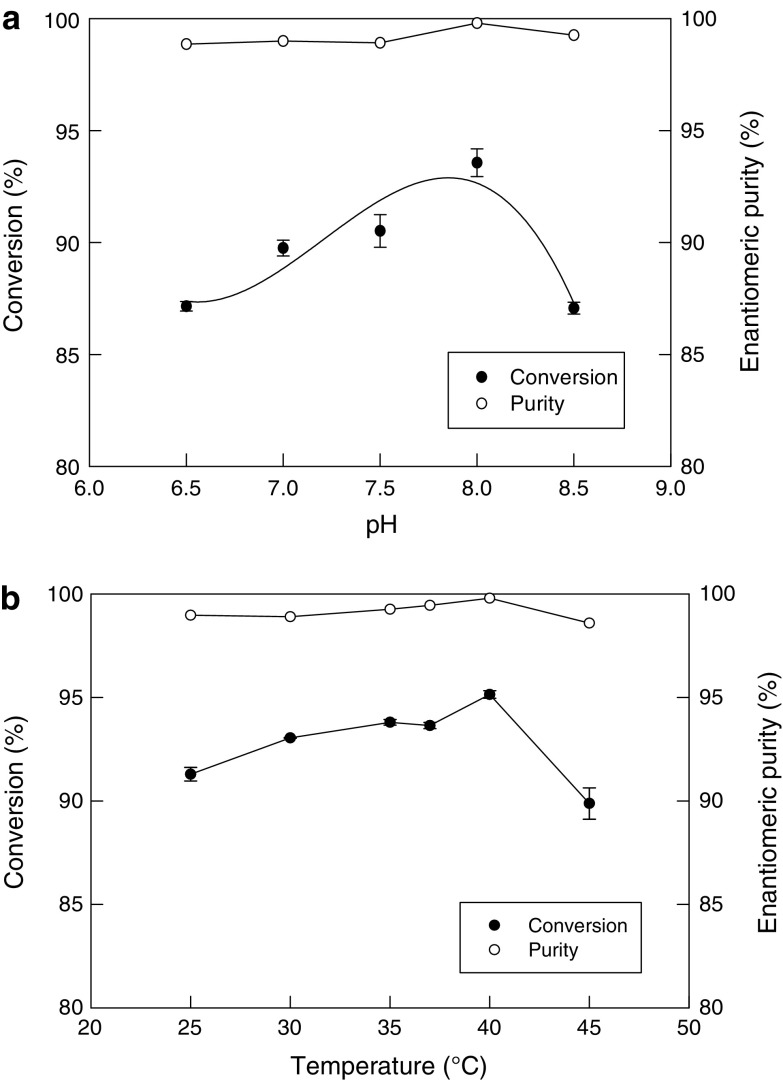
**a** Effect of pH on the conversion of mandelonitrile and the enantiomeric excess (purity) of the product. **b** Effect of temperature on the conversion of mandelonitrile and the enantiomeric excess (purity) of the product

In enantioselective hydrolysis of racemic mandelonitrile, the unreacted mandelonitrile undergoes spontaneous racemization via benzaldehyde and hydrogen cyanide formation. Generally racemization is favored at a slightly alkaline pH. Therefore, the effect of pH on substrate conversion was examined at various pH values ranging from 6.5 to 8.5. The optimal pH for maximizing the conversion was found to be 8 (Fig. 3a). The conversion was reduced both above and below the optimal pH value. The enantiomeric excess was always nearly 100 % and was not sensitive to pH in the range studied. The temperature for these studies was 37 °C.

As enzyme catalysis is quite sensitive to temperature, the effect of this variable on conversion was examined over the range from 25 to 45 °C. As shown in Fig. 3b, the maximum conversion value was observed at 40 °C. The other conditions for the reaction were an initial substrate concentration of 50 mM, a pH value of 8, 20 mg/mL cell loading in the beads, 1 g of beads per mL of reaction liquid, 4.3 mm bead size and 60 min of reaction time. The enantiomeric purity of the product was not particularly sensitive to the reaction temperature (Fig. 3b). It has been reported in the literature (Vejvoda et al. 2006) that polyvinyl alcohol and polyethylene glycol co-polymer were used to immobilize cells of *Fusarium solani* with 75 % recovery of nitrilase activity. pH optimum values shifted from pH 7 to pH 9–10 and the immobilized cells were very active at pH 11, while free cells at this pH showed negligible activity. The immobilization had no significant effect on the temperature optimum and thermostability of the nitrilase.

### Effect of substrate concentration

**Fig. 4:**
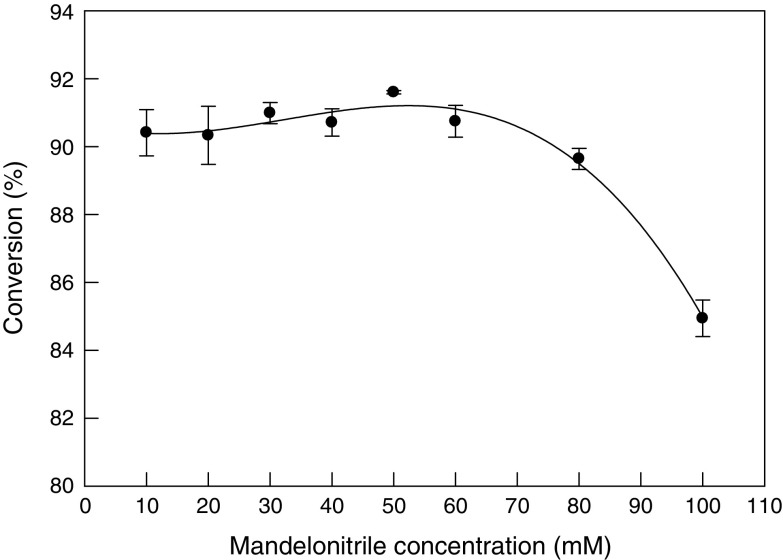
Effect of initial mandelonitrile concentration on the conversion

The effect of initial substrate concentration (10–100 mM) on conversion at 60 min (pH of 8, 40 °C) was determined using beads loaded in the reaction mixture. The cell loading was 20 mg/mL and the bead size was 4.3 mm. The conversion measured at 60 min was not too sensitive to the substrate concentration ≤50 mM (Fig. 4), but was progressively reduced as the substrate concentration was further increased. An excessive amount of substrate apparently inhibited the reaction. It is known that nitriles are toxic in nature and would limit the immobilized biocatalyst for the transformation reaction and there is a decrease in nitrilase activity due to the substrate inhibition (Zhang et al. 2011). A substrate concentration of 50 mM was therefore used in subsequent work, unless specified otherwise.

### Effect of cross linking treatment

**Fig. 5:**
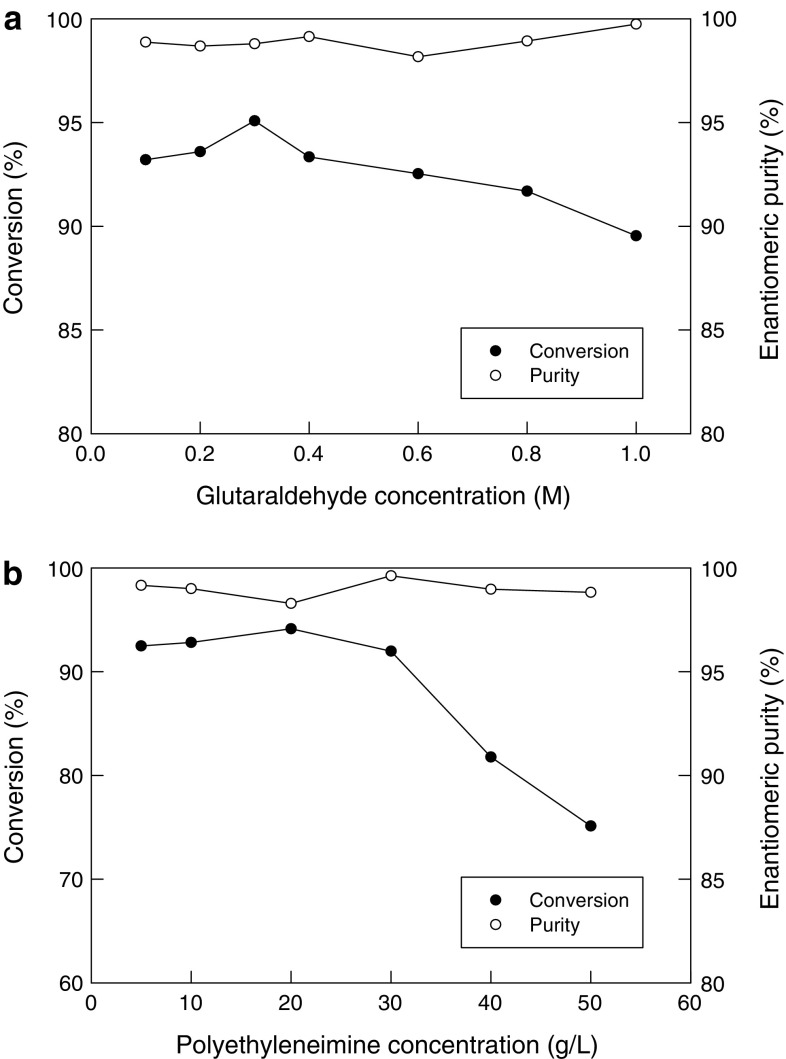
**a** Effect of glutaraldehyde crosslinker concentration on the conversion of mandelonitrile and the enantiomeric purity of the product. **b** Effect of combined treatment of cells with glutaraldehyde (0.3 M) and various concentrations of polyethyleneimine, on the conversion of mandelonitrile and the enantiomeric purity of the product

In attempts to further stabilize the biocatalyst beads, in separate experiments the beads (4.3 mm, made of 2 % sodium alginate) containing the immobilized cells (20 mg/mL) were further treated with various concentrations (0.1–1.0 M) of the crosslinking reagent glutaraldehyde (GA). For the cross linking, the beads were suspended in an aqueous solution of glutaraldehyde of a specified concentration for 2 h at room temperature. The beads were then washed thoroughly with water and used for the hydrolysis of mandelonitrile. The reaction conditions for the hydrolysis were 40 °C, pH 8, 60 min, cross-linked immobilized cells 20 mg/mL and an initial substrate concentration of 50 mM. Beads treated with 0.3 M glutaraldehyde achieved a substrate conversion of around 96 % (Fig. 5a), comparable with the conversion value [seen with otherwise identical beads that were not crosslinked (Fig. 3a, data at pH 8)]. The crosslinking treatment of the beads had no effect on the enantiomeric purity of the product formed (Fig. 5a).

In separate experiments, the alginate beads, as mentioned above, that had been pretreated with 0.3 M glutaraldehyde (2 h, room temperature) and thoroughly washed, were further treated with various concentrations (5–50 g/L aqueous solutions) of PEI for 2 h at room temperature. The washed beads were then used for the substrate conversion under the above-specified conditions. The conversion was about the same as for the control beads (i.e. treated with 0.3 M glutaraldehyde alone; Fig. 5a) so long as the concentration of the PEI during the second crosslinking treatment remained at ≤20 g/L (Fig. 5b).

### Reusability of catalysts

**Fig. 6:**
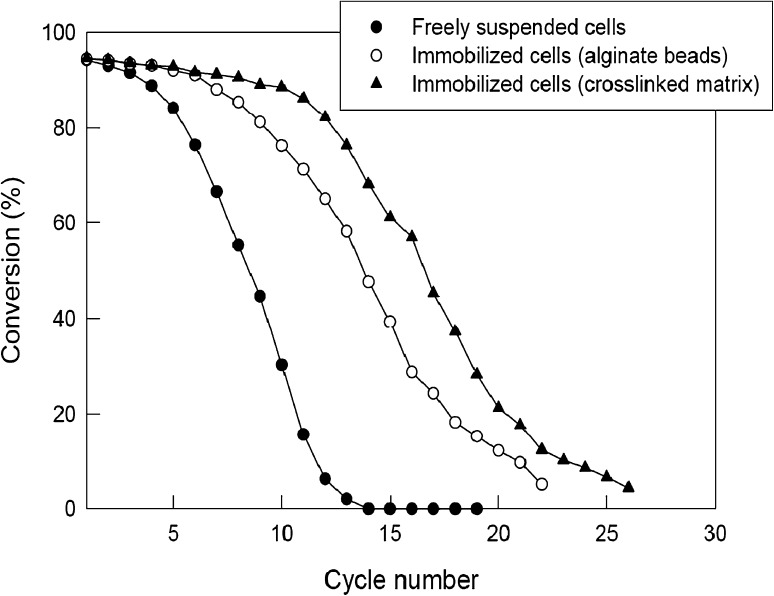
Effects of catalyst type and reuse cycle number on the conversion of mandelonitrile to mandelic acid

Reusability of freely suspended cells, the cells immobilized within alginate beads and the cells immobilized within glutaraldehyde (0.3 M) crosslinked alginate beads was tested by measuring the conversion at the end of each use cycle (1 h duration) of a repeated batch reaction. The reaction conditions were always: 40 °C, a pH of 8, 50 mM initial substrate concentration. After the reaction, the biocatalyst was recovered by centrifugation (for cells) or filtration (for beads), washed and reused for the next reaction cycle at the above-specified conditions. The concentration of the immobilized beads (20 mg cells per mL of bead volume) was always 1 g per mL of the reaction liquid. As shown in Fig. 6, the freely suspended cells rapidly lost activity. The alginate immobilized cells (no crosslinking) retained a high level of activity for around eight cycles of repeated use. The beads that had undergone the crosslinking treatment were somewhat more stable than the untreated beads (Fig. 6).

### Packed bed reactor

A packed bed reactor was used for further assessing the performance of the immobilized biocatalyst beads in continuous flow hydrolysis of mandelonitrile. The reactor had a working volume (i.e. nominal volume of the packed bed) of 75 mL and was made of a glass column with an internal diameter of 26 mm. The height of the packed zone was 200 mm. The reactor was jacketed for temperature control at 40 °C. The packing consisted of beads [cell loading of 20 mg/mL, 2 % concentration of sodium alginate, 4.3 mm beads, 100 g (fresh weight) of beads] of either the noncrosslinked catalyst or the catalyst that had undergone the crosslinking treatment with 0.3 M glutaraldehyde. An aqueous solution of substrate (50–500 mM, pH 8) was pumped in at the bottom of the vertically mounted reactor using a peristaltic pump. The feed flow rate was varied in different experiments. Samples were taken at the outlet to determine the steady-state conversion at various specified concentrations of the substrate in the feed.

### Effect of substrate feed flow rate

The conversion of the substrate at steady state was found to increase as the flow rate of the substrate increased in the range of 5–20 mL/h (data not shown). This was likely a result of improved diffusive mass transfer of the substrate to the biocatalyst beads. Further increase in substrate flow rate to up to 100 mL/h actually decreased conversion at an inlet substrate concentration of 50 mM as the residence time of the substrate in the reactor was insufficient for complete conversion to occur. These experiments used the non-crosslinked alginate beads as specified in the previous section. In view of these results, further experiments were carried out at a fixed feed flow rate value of 20 mL/h.

### Effect of substrate concentration in the feed

**Fig. 7:**
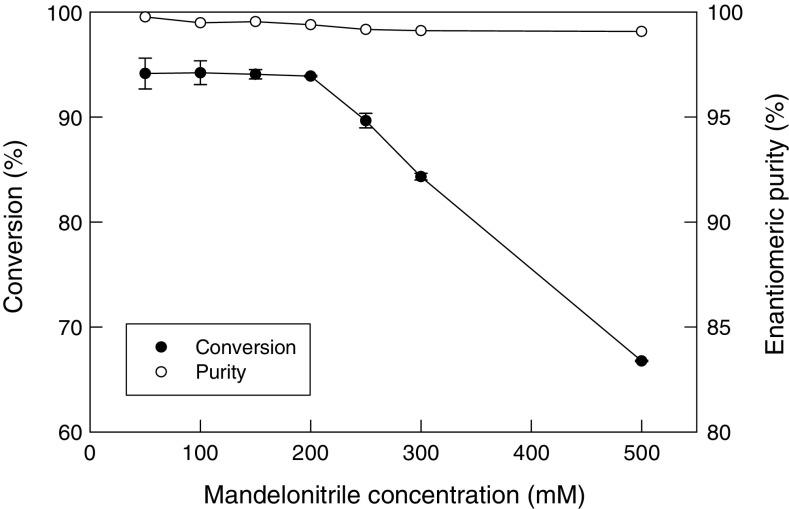
Effect of substrate concentration in the feed on the conversion of mandelonitrile and the enantiomeric purity of the product

The packed bed reactor with the non-crosslinked alginate bead catalyst was used under the above-specified conditions, but at different concentrations (50–500 mM) of the substrate in the feed. Conversion was measured at the exit once one bed volume of the feed had passed through after any change in the feed substrate concentration. The flow rate used (20 mL/h) and the nominal bed volume of 75 mL equated to a nominal residence time (based on the void volume of the bed) of 0.975 h assuming a cubic close packing of the spherical beads in the bed and therefore a void volume fraction of the bed of 0.26. Under these conditions, the substrate conversion was around 93 % irrespective of the substrate concentration in the feed so long as the concentration was in the range of 50–200 mM (Fig. 7).

Once the substrate concentration in the feed reached 200 mM, all the available catalyst in the bed was being used. Therefore, the conversion declined once the substrate concentration in the feed exceeded about 200 mM (Fig. 7) as an increasing proportion of the unconverted substrate left the bed. Nevertheless, compared with the shake flask studies in which the maximum concentration of the substrate was 50 mM at cell concentration of 20 mg/mL (Fig. 4), the packed bed could be operated at a fourfold higher substrate concentration possibly because of the higher spatial concentration of the catalyst in the bed.

### Effect of reactor reuse

**Fig. 8:**
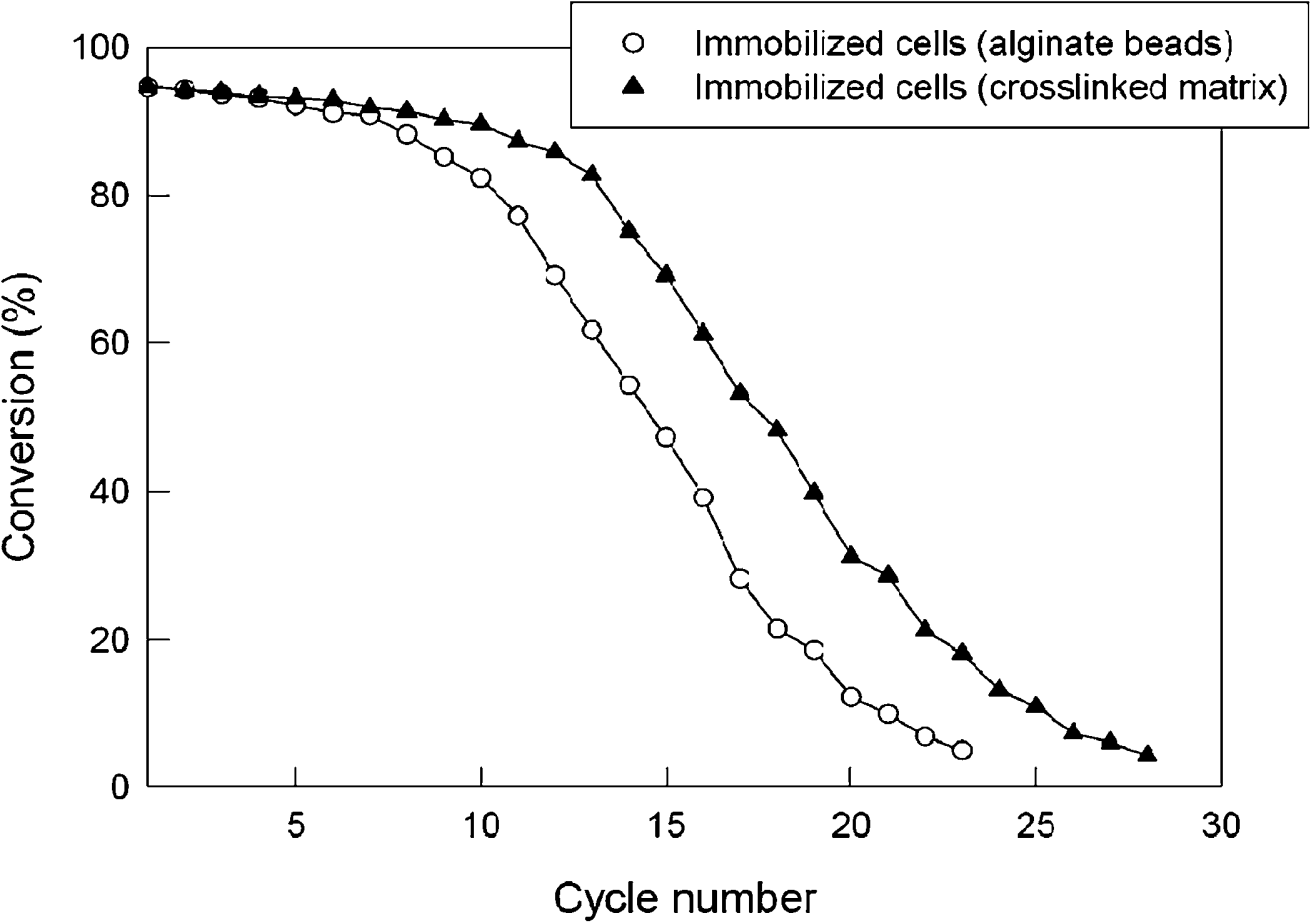
Effect of number of reuse cycles on the conversion of mandelonitrile by immobilized bead biocatalyst in the packed bed reactor

Reusability of the packed bed was tested by operating it continuously for 1 h at a flow rate of 20 mL/h and a substrate concentration in the feed of 200 mM. After each such operation, the bed was drained and the catalyst was washed. The next identical operational cycle was then carried out. The conversion was measured using samples collected at the exit of the reactor once a steady state had been attained. Both the immobilized beads and the immobilized crosslinked beads (0.3 M glutaraldehyde treated) were tested in separate experiments. As shown in Fig. 8, the immobilized bead catalyst could be used for nearly seven cycles without sacrificing the conversion. In contrast, the crosslinked bead catalyst could be used for up to 12 cycles without a significant degradation of activity. The results were generally similar to the ones seen in shake flasks (Fig. 6). It has been reported in the literature (Panova et al. 2007) that whole cells of recombinant *E. coli* expressing nitrilase from *A. faecalis* were immobilized in the κ-carrageenan beads crossed linked with glutaraldehyde and PEI and the beads were quite stable for at least 50 recycles. In other studies, alginate matrix was also used for the production of 3-hydroxyvaleric acid (Wu et al. 2007**)** and *p*-methoxy phenyl acetic acid (Chen et al. 2008). Stirred tank and packed bed reactors with the immobilized nitrilase adsorbed by hydrophobic and ion exchange interaction were also studied for the transformation reactions (Martínková et al. 2009). The matrix-encapsulated recombinant cells having nitrilase activity from *A. faecalis* were used for the synthesis of ammonium glycolate in CSTR with a conversion efficiency of 98–99 % (Panova et al. 2007).

## Conclusions

Alginate bead catalysts of immobilized cells of a nitrilase-containing recombinant *E. coli* could be used successfully for stereo-selective hydrolysis of mandelonitrile to (*R*)-(−)-mandelic acid. Under optimal conditions (40 °C, pH 8, 20 mg cells per mL of beads) the conversion at 60 min exceeded 90 % both in suspensions of the catalyst (1 g/mL beads concentration) and in packed beds containing it. In the packed bed, a substrate feed concentration of up to 200 mM could be used at a flow rate of 20 mL/min (residence time of 58.5 min) while attaining a conversion of nearly 93 %. The enantiomeric purity of the product was always close to 100 %. The packed bed could be used repeatedly for up to 12 cycles of operation (1 h duration per cycle) without a significant loss of the ability to convert the substrate.
